# Serotonin is a gap junction-permeable neuronal tracer in the mouse retina

**DOI:** 10.3389/fopht.2023.1151024

**Published:** 2023-03-22

**Authors:** Gergely Szarka, Gyula Hoffmann, Tamás Kovács-Öller, Béla Völgyi

**Affiliations:** ^1^ Szentágothai Research Centre, University of Pécs, Pécs, Hungary; ^2^ Department of Comparative Anatomy and Developmental Biology, University of Pécs, Pécs, Hungary; ^3^ Center for Neuroscience, University of Pécs, Pécs, Hungary; ^4^ NEURON-066 Rethealthsi Research Group, Pécs, Hungary

**Keywords:** gap junction, electrical synapse, ganglion cell, amacrine cell, Neurobiotin, serotonin, retina

## Abstract

**Introduction:**

Gap junctions are dynamically modulated bridges allowing the transcellular passage of ions and small molecules with a molecular mass of up to 1 kDa, a mechanism utilized for molecular communication purposes by living cells. This same mechanism is also exploited by scientists to reveal the existence of gap junction contacts by the cell-to-cell movement of tracers. However, multiple labeling experiments require the availability of multiple gap junction-permeable tracers.

**Methods:**

To this end, we utilized the well-known transient OFF alpha retinal ganglion cell (RGC)-coupled array as a model system to study and compare the transjunctional movement of neurobiotin (NB), a commonly used tracer, and serotonin, a recently identified tracer.

**Results:**

Although the transjunctional movement of serotonin has been established in cell cultures, here we show, for the first time, that serotonin is also a potent tracer in *in vitro* tissue. In addition, serotonin is lighter than the classical gap junction-permeable NB, and thus, we expected that tracer movement would be comparable to or better than that of serotonin. We found that intracellular serotonin injections result in the labeling of the coupled transient OFF alpha RGC array very similar to those of the classical NB-labeled arrays. Both serotonin and NB-injected transient OFF alpha RGCs displayed the well-known pattern with coupled RGCs and a cohort of coupled wide-field amacrine cells (ACs).

**Discussion:**

By using morphological characteristics, we confirm that the serotonin and the NB-coupled AC arrays are identical, and thereby confirm that serotonin is a potent gap junction-permeable tracer and can be readily used as an alternative to NB in *in vitro* tissue. Moreover, serotonin can be utilized in parallel with other dyes or tracers, enabling the use of multiple labels in the same material.

## 1 Introduction

It has been shown that electrical synapses (gap junctions) permit the transcellular diffusion of ions and low-molecular-weight compounds typically lighter than 1 kDa. Researchers over the past four decades have exploited lightweight dyes and tracer molecules to reveal gap junction connections between neurons of the nervous system, including cells in the vertebrate retina. Initial studies utilized fluorescent dyes such as Procion Yellow or Lucifer Yellow, but these experiments resulted in limited transjunctional diffusion owing to the relatively large size of the molecules and revealed only gap junction connections with exceptionally high transjunctional conductance, as in retinal horizontal cells (1, 2), whereas low-conductance gap junction connections remained undetected. The real breakthrough started with the discovery of biocytin and its derivatives, including neurobiotin (NB), which, thanks to their low weight (*M*_biocytin_ 372 g/mol; *M*_NB_ 322 g/mol), were able to pass through even the low-conductance inner retinal gap junctions (3–6).

Although the solutes of these compounds are not fluorescent, and they, therefore, fail to mark the primarily stained cell directly, their presence can be revealed by the use of the appropriate *post hoc* streptavidin histochemistry. In addition, horseradish peroxidase/diaminobenzidine-based histochemistry can be used to reveal NB staining in samples for transmission electron microscopy. The domination of NB as the most popular gap junction-permeable tracer has not declined, but a few additional tracer candidates have emerged in recent years. A seminal work by Mills and Massey, for example, provided evidence that a series of other biotinylated tracers can also be utilized (7); however, all members of this particular molecular arsenal are heavier than NB, thus reducing (or entirely impeding) their gap junction permeability. Moreover, like NB, these tracers are visualized by the same streptavidin histochemistry, and thus, they cannot be utilized for dual or multiple labeling alongside NB.

The discovery of the gap junction-permeable fluorescent tracer PO-PRO-1 (8) allowed for both multiple labeling (e.g., in combination with NB) and the direct visualization of injected neurons. However, the water solubility of PO-PRO-1 is satisfactory only after dissolving it in dimethylsulfoxide, thus hindering its use in functional studies. Alexa Fluor™ hydrazide dyes (ThermoFisher Scientific, Waltham, MA, USA) have been used as neuronal dyes to fill cells following electrophysiological examinations. Some Alexa Fluor dyes have been reported to diffuse from one cell to its neighbors in a gap junction-coupled array (9); however, their utilization seems to be restricted to only some of the dyes (Alexa Fluor 488 hyrazide) and to certain coupled arrays with relatively high gap junction conductivity [connexin (Cx) 45-coupled wide-field amacrine cells (ACs)]. While a generally applicable fluorescent gap junction-permeable tracer is yet to be discovered, another potential tracer (serotonin) has been tested. Recently (10), serotonin has been shown to pass between gap junction-coupled cells in cultures with sensitivity and mobility comparable to those of NB. However, other than in cell cultures, serotonin has not been tested in nervous tissue *in vitro*. To fill this gap, we used serotonin as a potential gap junction-permeable tracer in *in vitro* mouse retina preparation. We investigated the well-studied OFF alpha subtype of retinal ganglion cells (RGCs) that has been shown to maintain two sets of gap junctions, one connecting neighboring RGCs to a homologous RGC array and another that serves the communication of OFF alpha RGCs with nearby wide-field Acs (11–13). While this latter connection has been shown to depend on Cx36 protein subunits, the direct RGC–RGC connections likely comprise some, yet unidentified, Cx molecules (12, 14–16). In addition to their well-known gap junction-coupling pattern, OFF alpha RGCs are perfect candidates for systematic *in vitro* experimental studies, as their characteristic soma shape and size allow the experimenter to target them easily, even in the unstained preparation. In addition, the coupled OFF alpha RGC arrays have already been tested for the permeability of NB (11–13, 16, 17), Lucifer Yellow (18–20), and PO-PRO-1 (8), and have also been studied in pharmacological gap junction blockade studies (15, 21–23).

Here we follow up on this line of studies and provide further details on the gap junction-mediated molecular exchange between cells in the coupled OFF alpha RGC array. In this scheme, the delivery of either the classical tracer NB or the lower-molecular-weight alternative, serotonin, was tested. We found that serotonin injections into OFF alpha cells reveal a tracer-coupled array highly similar to those of the NB-stained arrays. This, therefore, indicates that serotonin is a potent alternative to NB as a transjunctional tracer. We also revealed an interference mechanism that impedes the movement of tracers across gap junctions when they are used in multiple tracer injections. This tracer interference was robust when tracers were injected from the same glass pipette [shown for Alexa Fluor 568 hydrazide dye (Alexa568) and NB] and also appeared when tracers were injected consecutively from separate micropipettes (shown for serotonin and NB). Altogether, serotonin in our hands proved to be a potential gap junction-permeable tracer that can be utilized in future *in vitro* studies of the vertebrate retina and, most likely, in studies of other brain structures.

## 2 Materials and methods

### 2.1 Animals and preparation

Adult mice (P30–90) from the *Thy1*-GCaMP3 mouse line (Jax, Strain #:017893) were used to target transient OFF alpha cells, which are recognizable because of their large soma and dendritic arborization. The mice were maintained in a 12/12 hours dark/light cycle; all experiments were carried out during the day, with dissections between 10 and 12 a.m. and after 12 hours of dark adaptation prior to experiments. The mice were deeply anesthetized with the inhalation of Forane (4%, 0.2 mL/L) and were then sacrificed using cervical dislocation. Eyes and retinas were removed under dim red illumination and hemisected anterior to the ora-serrata. The cornea, lens, vitreous humor, and pigment epithelium were isolated, and the resultant isolated retina was attached to a filter paper (Millipore, Merck KgaA, Darmstadt, Germany). Specimens were then placed in a superfusion chamber, mounted in a light-tight Faraday cage, and rinsed with an oxygenated (95% O_2_, 5% CO_2_) and heated (34°C) mammalian Ringer solution (pH 7.4). Animal handling, housing, and experimental procedures were reviewed and approved by the ethics committee of the University of Pécs (BA/35/51-42/2016 and BA02/2000-69/2017). All animals were treated in accordance with the Association for Research in Vision and Ophthalmology Statement for the Use of Animals in Ophthalmic and Vision Research. All efforts were made to minimize pain and discomfort during the experiments, and all procedures were carried out by obeying the 3R law.

### 2.2 Patch clamp recordings, dye injections

Patch clamp recordings were performed with an Axopatch 200B patch clamp (PC) amplifier (Axon Instruments Inc., Union City, CA, USA) and ECS-filled PC pipettes (≈20 MΩ; borosilicate glass, 1.5/0.84 mm ID/OD, WPI) in loose patch configuration (voltage clamp mode). Signals were digitized with a Digidata 1440A ADC (Axon Instruments, Inc.) and acquired with WinWCP software (John Dempster, University of Strathclyde, Glasgow, UK). Electrodes used for dye staining and stimulation were filled with ICS (20–30 MΩ borosilicate glass pipettes, 1.5/0.84 mm ID/OD, WPI). Electrodes were pulled with a P-87 micropipette puller (Sutter Instruments, Novato, CA, USA). The ICS contained 125 mM potassium gluconate, 8 mM NaCl, 0.6 mM MgCl_2_, 1 mM EGTA, 10 mM HEPES, 2 mM Mg-ATP, and 0.4 mM Na-GTP at pH 7.3 (KOH adjusted) and was supplemented with a combination of 0.5% A568-hydrazide and either 4% NB or 0 1% serotonin to fill up target cells *via* electroporation [NB: +65 mV pulses at 1 Hz (*V* = –50 mV; *R* = 90 MΩ); serotonin: –65 mV pulses at 1 Hz (*V* = 15 mV; *R* = 90 MΩ)].

### 2.3 Light stimulation

Light stimuli patterns were programmed in the PsychoPy free cross-platform software (24) and were then delivered by a high-definition LED projector through an ND2 filter and directly focused on the surface of the retina. To verify the identity of the transient OFF alpha cells, full-field and approaching stimuli were utilized. For full-field stimuli, full-field white (gv: 256) and black illumination (gv: 0) were alternated (cycle: 1 s, with 0.5 s white illumination and 0.5 s black illumination). For the approaching stimuli, a 40-μm black circle was projected over soma for 1 s. The diameter was then increased from 40 μm to 240 μm in 0.5 s (400 μm/s).

### 2.4 *Post hoc* histochemistry

To visualize NB-filled cells, samples were incubated for a minimum of 30 min. Tissues were then fixed in 4% paraformaldehyde solution for 15–25 min, washed with phosphate buffered saline (PBS), and blocked with CTA (5% Chemiblocker, 0.5% Triton X-100, 0.05% sodium azide in PBS) overnight, then incubated in Streptavidin Cy5 (Thermo Fisher Scientific; 1,500× dilution) in CTA overnight. Samples with serotonin-filled cells were processed for serotonin immunohistochemistry by applying anti-serotonin antiserum (Sigma-Aldrich, St. Louis, MO, USA, catalog number: S5545, 2,000× dilution) for 2 days and, following a thorough washing (4× in PBS), adding DyLight™ 405 AffiniPure Goat Anti-Rabbit (111-475-003) secondary serum to the tissue for fluorescent visualization. Washed retina samples were placed on slides mounted in VectaShield (Vector Laboratories, Newark, CA, USA) and cover-slipped for microscopy. In addition to tracer visualization, we also performed other immunohistochemistry experiments with the same protocol as the serotonin labeling for rabbit choline acetyltransferase (ChAT; ThermoFisher, PA5-29653, 1,500×) and rabbit hyperpolarization-activated cyclic nucleotide-gated potassium channel 4 (HCN4; Alomone Labs, Jerusalem, Israel; APC-052, 1,500×).

### 2.5 Confocal microscopy and image processing

Retinal samples were scanned with a Zeiss LSM710confocal microscope with 20× (*Z* = 1 μm; Zeiss W Plan-Apochromat 20/1.0) and 63× objectives (*Z* = 0.5 μm; Zeiss Plan Apochromat 63/1.4) at high resolution and normalized laser intensity. Minor manipulations of brightness and contrast of images were performed in FIJI – ImageJ, NIH, and Adobe Photoshop CC (Adobe Systems Inc., San Jose, CA, USA).

### 2.6 Injected cell array reconstructions

Confocal z-stacks were imported into FIJI. After utilizing the Simple Neurite Tracer (25) plugin, all paths of each cell were traced. First, the soma was traced in concentric circles from the first to last virtual section in which it was present, then, originating from the soma path, further paths were traced to the upcoming intersection until the whole arbor was selected. Having selected all the paths belonging to each cell, the fill-out feature was performed, and the fill was exported as a grayscale image. The grayscale images were used for further analyses and, using the FIJI three-dimensional (3D) viewer plugin, 3D models were generated for visualization.

## 3 Results

### 3.1 Identification of transient OFF alpha ganglion cells in the mouse retina

**Figure 1:**
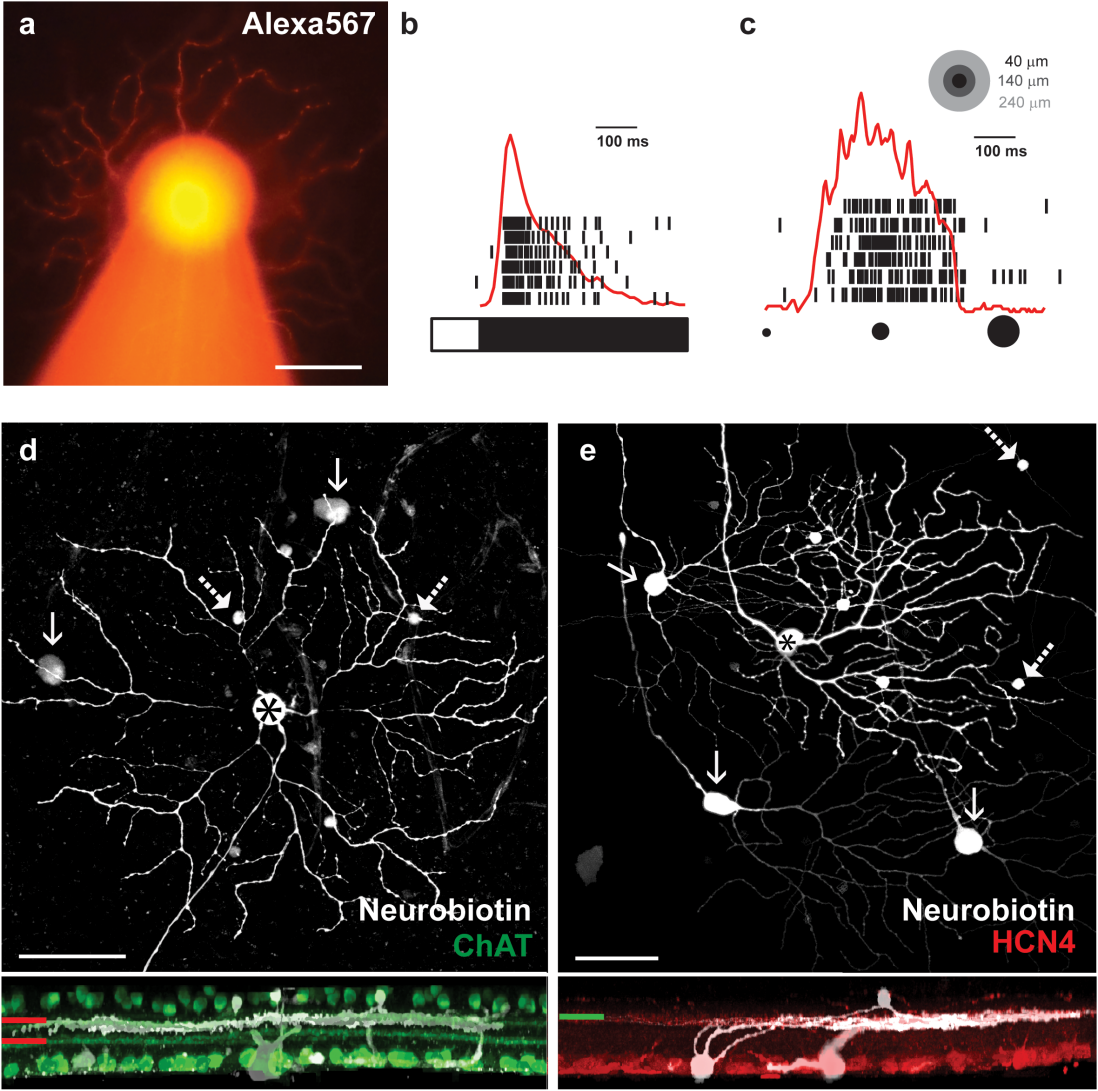
Identification of transient OFF alpha RGCs in the mouse retina. **(A)** Light microscopic image of an Alexa568-labeled OFF alpha RGC filled with a borosilicate glass pipette (visible as an out-of-focus structure at the bottom of the panel). **(B)** Spiking response of the alpha RGC (shown in panel **A**) to photopic full-field light stimuli. The bar on the bottom represents the duration (white) and the offset (black) of the stimulus, whereas the time stamps above represent individual responses of the targeted OFF alpha cell to six consecutive trials. The overlaid red curve is the peristimulus time histogram (PSTH) generated on the corresponding spike responses. **(C)** Spiking activity (black time stamps) and PSTH (red curve) of the OFF alpha RGC as a response to an approaching stimulus – a dark spot with an increasing diameter (from 40 μm to 240 μm in 0.5 s, equivalent of a 400 μm/s approaching movement, represented by the spots in the top right corner. **(D)** Face (top) and side (bottom) views of the result of the NB injection of the transient OFF alpha RGC (asterisk). The injected RGC and tracer-coupled nearby ACs (dashed arrows) and RGCs (arrows) are shown in white, whereas ChAT counter-labeled starburst cell processes and cell bodies are green. The ChAT-positive processes demarcate strata 2 and 4 (red lines to the left), and thus, the stratification of injected and coupled neuronal processes (white) could be determined. Dendrites of stained neurons are clearly located right below the stratum 2 OFF starburst processes. **(E)** Face (top) and side (bottom) views of another NB-injected transient OFF alpha RGC (asterisk). The injected RGC and tracer-coupled nearby ACs (dashed arrows) and RGCs (arrows) are shown in white, whereas HCN4 counter-labeled type 3a OFF bipolar cell axon terminals are red (the green line represents the IPL stratification level). The HCN4-positive processes clearly costratify with dendrites of the injected and coupled neuronal process (white). Scale bars in all images: 50 μm.

The first line of experiments was carried out using the *Thy1*-GCamP3 mouse line, in which the GCamP3 construct is expressed under the control of the Thy1 (CD90, thymocyte 1 surface antigen) promoter, which labels approximately 70%–75% of all RGCs (26). First, the combination of Alexa568 and NB was injected into some (16 injected cells in six mice) large and bright RGC somata in the *in vitro* retina preparation under epifluorescent light (Λ = 488 nm). The Alexa568 staining provided immediate feedback that the injected cell displayed the characteristic transient OFF alpha RGC soma/dendritic morphology (**Figure 1A**; note that all injections were performed in transient OFF alpha RGCs in this study, but for simplicity, we refer to them as OFF alpha RGCs on several occasions). In addition to the Alexa568 staining, we were also guided by the transient OFF polarity light responses of targeted cells to a full-field stimulation as well as an attenuated and prolonged spiking as a response to an approaching stimulus (**Figures 1B, C**). Finally, we utilized *post hoc* immunocytochemistry for either ChAT or HCN4 to mark specific layers in the inner plexiform layer (IPL). The ChAT staining labels starburst ACs, whose narrowly stratified dendrites demarcate strata 2 and 4 in the IPL, provided us guidance for the precise stratification of injected and tracer-coupled cells. In addition to the ChAT labels, we also tested whether the injected RGCs co-stratified with the axon terminals of HCN4-positive type 3a OFF cone bipolar cells, another OFF alpha RGC characteristic (27) (**Figures 1D, E**). Only cells whose identity could be unequivocally confirmed by both the *in vitro* and *post hoc* histochemistry clues previously listed were utilized in the rest of this study.

### 3.2 NB and serotonin tracer coupling of OFF alpha RGCs

**Figure 2:**
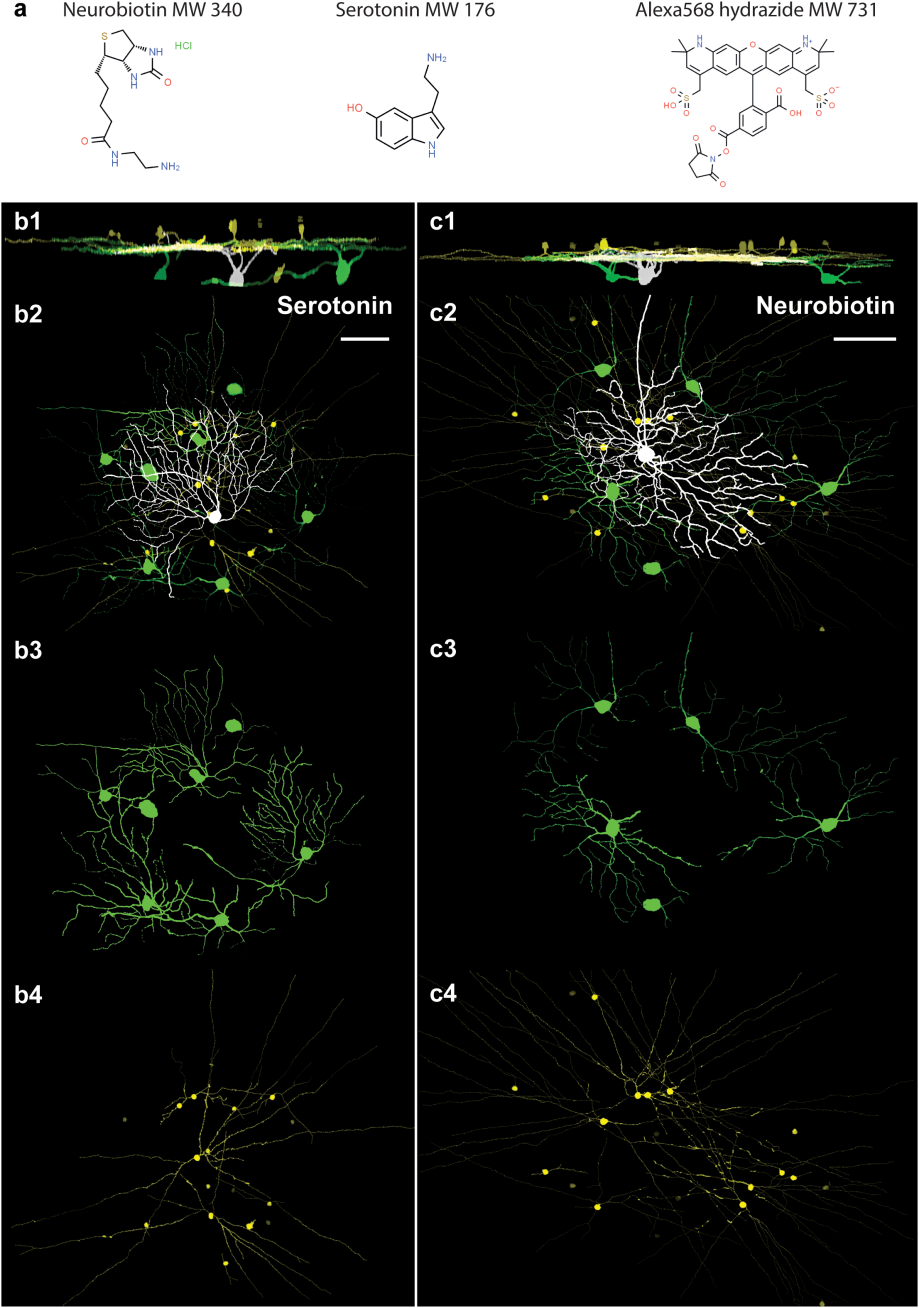
Comparison of serotonin and NB coupling of OFF alpha RGCs in the mouse retina. **(A)**. The chemical structures and molecular weights (MWs) of NB, serotonin, and Alexa568 compounds that were used in this study (Chemspider.com). **(B)**. 1–4. Side (1) and face (2–4) views of serotonin-injected OFF alpha RGCs (white). Serotonin-coupled nearby RGCs (green) display a dendritic stratification level and morphology resembling the injected OFF alpha RGC. The somata and processes of serotonin-coupled ACs (yellow) display a wide-field morphology. **(C)**. 1–4. Side (1) and face (2–4) views of NB-injected OFF alpha RGCs (white). Coupled nearby RGCs (green) display a dendritic stratification level and morphology resembling the injected OFF alpha RGCs. In line with previous descriptions (11, 12), the somata and processes of NB-coupled ACs (yellow) display a wide-field morphology. Scale bars: 50 μm.

In the first experimental design, we injected neighboring cells with either the classical neuronal tracer NB (*n* = 11; MW = 322.8 g/mol; **Figure 2A**) or the even smaller serotonin (*n* = 5; Mw = 176.215 g/mol) intracellularly into OFF alpha RGCs and revealed the coupling patterns *via post hoc* histochemistry. It has previously been reported that OFF alpha RGCs injected with NB display a characteristic tracer coupling pattern, including direct coupling to neighbor alpha RGCs and a cohort of nearby ACs (11, 13–15, 28). The tracer coupling patterns of the targeted RGCs in this study corresponded perfectly with previous observations of NB-coupled OFF alpha RGC arrays (**Figure 2B**). In a second cohort of injections, we replaced NB with serotonin in the internal solution. Serotonin has been shown by Hou and colleagues (10) to be a potentially useful gap junction-permeable tracer in cell cultures. However, to the best of our knowledge, serotonin has not previously been used as a neuronal tracer in *in vitro* tissue. In our study, following serotonin injection, the OFF alpha RGC targets were perfectly stained by the tracer and the full soma/dendritic morphology of the injected cells could be revealed. Based on the serotonin labels, our injected RGCs displayed an OFF alpha cell morphology identical to that of cells labeled with NB (**Figure 2C**). In addition to elucidating the morphology of the injected cell, the serotonin label also revealed serotonin-coupled direct neighbor RGCs and a cohort of ACs. Therefore, the serotonin and NB tracer coupling patterns of OFF alpha RGCs appeared identical for the initial investigations.

### 3.3 Labeling interference of parallel injected tracers

**Figure 3:**
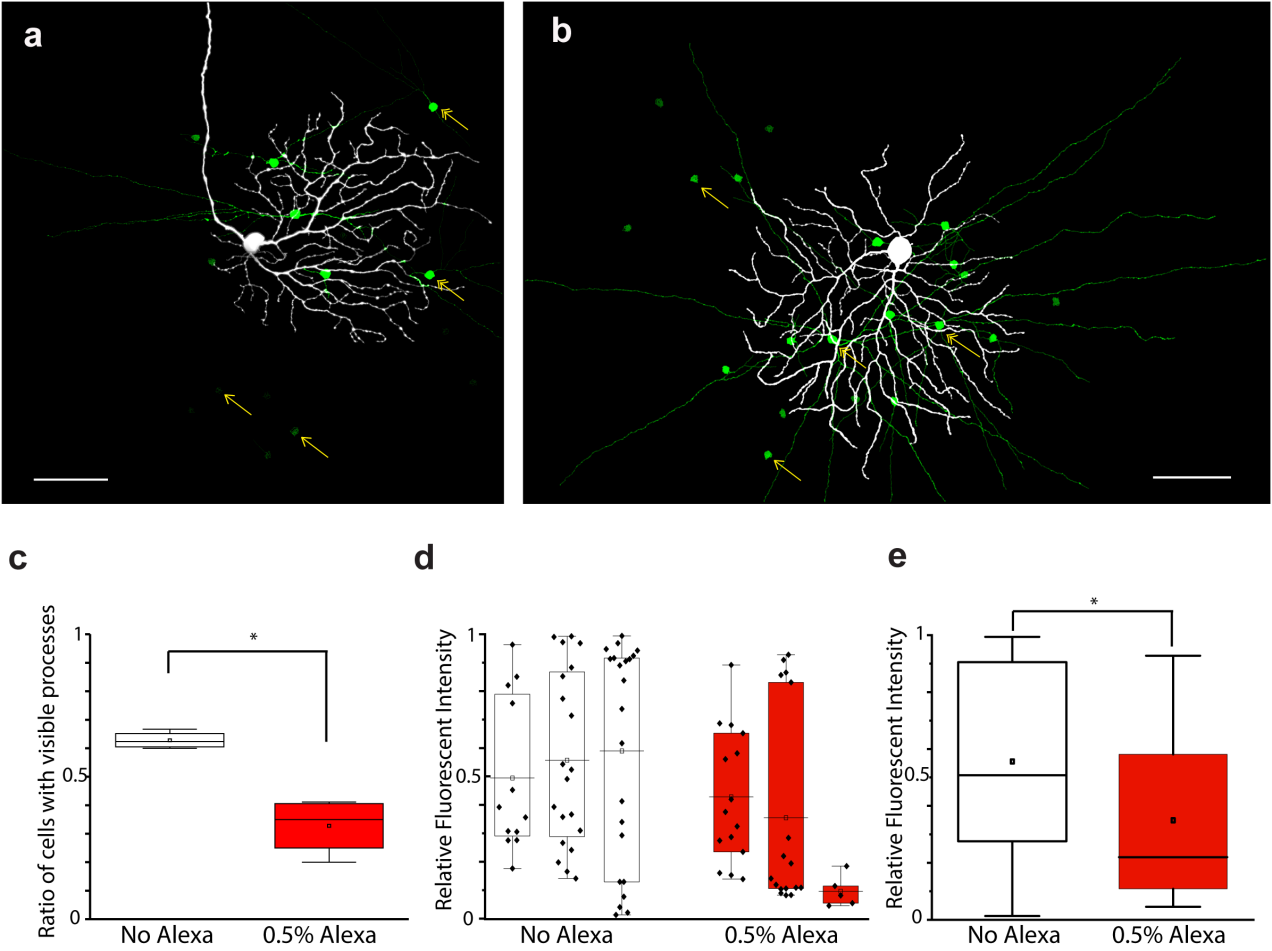
Interference in the diffusion of tracers/dyes injected simultaneously in the OFF alpha RGC array. **(A, B)** Images showing the results of OFF alpha RGC NB injections with **(A)** and without **(B)** Alexa568 (0.5%) in the internal solution. **(C)** Diagram displaying the ratio of coupled ACs and showing P (compared with the total number of coupled AC somata in the corresponding injection) with or without Alexa568 in the internal solution. Statistical analysis showed a significant (*p* < 0.05; asterisk) difference in the number of coupled ACs with or without Alexa568 in the pipette. **(D)** Diagram showing the relative brightness of coupled AC somata in 3 + 3 NB-injected OFF alpha RGC arrays with or without Alexa568. **(E)** Summary diagram showing the averaged brightness of coupled ACs of the 3 + 3 tracer injections shown in panel **(D)** Statistical analysis revealed a significant (*p* < 0.05; asterisk) difference in brightness between ACs with and those without Alexa568 in the pipette. Scale bar: 50 μm.

Next, we performed dual tracer injections by dissolving both NB and serotonin in the internal solution to see whether the two tracers would mark a partially overlapping neuron population for the same RGC array. However, all these trials failed to deliver a tracer-coupled array as extensive as those of the previous injections in which either NB or serotonin was utilized alone. In fact, both tracer labels were unacceptable for further investigations of RGC coupling (not shown). The aforementioned experiments suggested that tracer molecules interfere with each other when they are injected simultaneously from the same glass pipette. It was unclear, though, whether this interference was due to an NB/serotonin-specific mechanism, or whether it was a more general phenomenon. To test this, we performed serotonin and NB injections (*n* = 3 and *n* = 5, respectively) into OFF alpha RGC targets, but this time, we omitted the Alexa568 that guided us during the *in vitro* manipulation under the fluorescent view (**Figure 3A**). This introduced an extra uncertainty into visual neuron targeting, but using the rest of the clues (i.e., soma morphology under the differential interference contrast microscopy (DIC), light responses, *post hoc* histochemistry), we were able to confirm that our injected neurons were in fact OFF alpha RGCs. We analyzed these NB-injected arrays and, to our surprise, the reduced concentration of Alexa568 resulted in a brighter and more extensive NB labeling of the injected OFF alpha RGCs (**Figure 3B**) than the injection of both Alexa568 and NB together. NB, when injected alone, stained a higher ratio of ACs with visible processes (P) [m_NB_ = 0.63 ± 0.03 (SD), *n* = 5; m_NBAlexa_ = 0.32 ± 0.09, *n* = 5) and the relative AC soma staining intensity was also higher (m_NB_ = 0.53 ± 0.31, *n* = 69; m_NBAlexa_ = 0.24 ± 0.31, *n* = 27) than when injected with Alexa568. In addition, the labeling of the coupled RGCs appeared stronger (a quantitative analysis was not performed) in cells injected only with NB, and their full dendritic structure was very often revealed (**Figures 3C–E**). Interestingly, this phenomenon was present in both in cells injected with NB and Alexa568 and in cells injected with NMB and serotonin, even though the iontophoresis of these tracers is done by opposing charges. This set of experiments, together with previous observations on NB/serotonin dual injections, indicated that tracers interfere with each other when injected in parallel, and this interference is likely a general phenomenon and not tracer specific because it occurred in both NB/Alexa568 and NB/serotonin injections.

### 3.4 A single subtype of coupled ACs is revealed in both serotonin and NB-injected OFF alpha RGC arrays

Although the results of serotonin injection of alpha RGCs of (above) resembled the well-known results of NB injections, the somata of some serotonin-coupled ACs appeared somewhat smaller and more lightly stained than those in NB injections. However, this apparent discrepancy could be explained by the dissimilar labeling strength, and thus, a more elaborate, quantitative examination was necessary to reinforce (or reject) these qualitative observations. According to the classical descriptions, OFF alpha RGCs of the mouse retina display NB coupling to two cohorts of ACs with somata in the inner nuclear layer. One population has been reported to display medium-sized round somata and strong NB staining, whereas cells in the second population have relatively small somata and lighter staining, and the soma shape is often fusiform or triangular (11–13). The first AC population also displayed long straight dendritic processes that branched rarely, thus showing a typical wide-field AC morphology (we refer to this cell as type 1-coupled AC in this paper). However, dendritic staining of the putative second AC population has not previously been detected in any studies (we refer to this cell as type 2-coupled AC in this paper).

**Figure 4:**
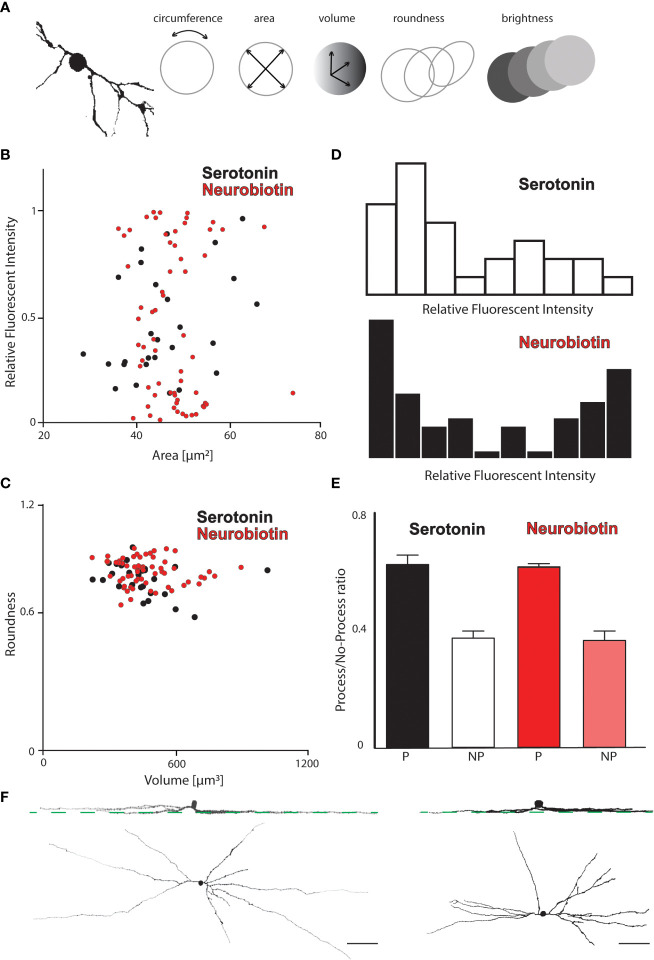
Morphometric analysis of tracer coupled ACs in the OFF alpha RGC array. **(A)** Schematic drawings show that the soma and primary dendrites of a reconstructed AC appeared coupled to an OFF alpha RGC (left) and display the measured morphologic features (circumference, area, volume, roundness, and brightness) of coupled AC somata. **(B)** Histogram displaying the fluorescent intensities of tracer-coupled ACs in the serotonin-injected (black) and NB-injected (red) OFF alpha RGC arrays as a function of the soma area (pooled data from four serotonin and five NB OFF alpha cell injections). While both tracers labeled ACs with various soma sizes equally, the fluorescence intensity appears to distinguish between a bright and a dim AC population in both injections. **(C)** Histogram displaying the roundness of tracer-coupled ACs in the serotonin-injected (black) and NB-injected (red) OFF alpha RGC arrays as a function of the soma volume. Neither serotonin- nor NB-coupled AC somata could be further divided based on these two characteristics. **(D)** Histograms show the frequency of AC somata with various relative fluorescence intensities (relative to the brightness of the injected OFF alpha RGC) in serotonin-injected (top) and NB-injected (bottom) arrays. AC somata in both injected arrays seem to be separated into dimmer (left side) and brighter (right side) somata; however, this distinction between the two populations was more obvious in the NB-injected arrays, while serotonin-stained coupled ACs more homogeneously. **(E)** The result of an analysis in which AC somata were sorted into two subpopulations depending on the presence (P) or absence (NP) of visible processes. Values of P and NP AC somata are normalized to the total number of coupled ACs in a certain array (P + NP) to compare values across injections. Based on the data, serotonin and NB showed no difference in the staining of coupled ACs. In both injections, ≈60% of the coupled ACs displayed some dendritic processes, while for the rest (≈40%), only the somata were visible. **(F)** Panels showing face and side views of ACs that appeared to be tracer coupled to either NB- (left) or serotonin-injected (right) OFF alpha RGCs. Green dashed lines mark the stratification level of HCN4-positive type 3a bipolar cell axon terminals in the IPL. Scale bar: 50 µm.

Here, we performed morphometric measurements on AC somata with two goals: (i) to find quantitative support for the existence of the two AC populations based on basic somatic features, and (ii) to find any dissimilarity between serotonin and NB AC coupling and/or tracer preference. We performed these measurements in four serotonin- and five NB-injected OFF alpha RGC arrays, which resulted in 37 serotonin- and 69 NB-labeled ACs in total. The measured parameters included (i) soma diameter, (ii) soma circumference, (iii) soma volume, (iv) labeling intensity (relative to the injected RGC), and (v) roundness (note that diameter, circumference, and volume are not independent parameters, and their mutual relations were, therefore, not examined) (**Figure 4A**). The findings of these measurements and the consecutive data analysis were twofold. First, AC somata largely separated into two populations whenever brightness was plotted against any other features (**Figure 4B**). However, ACs formed a single population when any permutation of the soma size (diameter, circumference, and volume) and roundness were plotted (**Figure 4C**).

These results, therefore, indicated that labeling intensity (brightness) is the only soma feature to clearly separate ACs into two populations. This finding was equally true for labeled ACs in both serotonin and NB injections. Frequency analysis of coupled AC soma brightness appeared to reveal two populations, one with brighter somata and another with dim staining following both serotonin and NB tracer injections (**Figure 4D**). The only discrepancy between the two tracer labels is the relative homogeneous staining of coupled AC somata in serotonin injections, which resulted in a less complete separation of the two AC subpopulations in the diagram. In addition to the above quantitative measures, we also registered whether visible dendritic processes could be detected for the coupled AC somata. We found coupled ACs either with P or with no sign of processes (NP) both in cells injected with serotonin and in those injected with NB. When the numbers of the P and NP populations were compared, we found no difference in the ratios of P and NP ACs between serotonin- and NB-injected cells (**Figure 4E**); ≈60% of the tracer-coupled ACs displayed dendritic staining, whereas, in the remainder, only the somata were visible. The close similarity of the AC labels shows that serotonin and NB, when injected into OFF alpha RGCs, couple the same set of ACs, and therefore, the OFF alpha RGC/AC gap junctions show no signs of a preference for one of the two examined tracers. In addition to carrying out the above quantitative analysis, we also studied the dendritic morphology of ACs (only the P population) and found that the NB- and the serotonin-injected arrays appeared very similar (**Figure 4F**). The similarities included (i) the regularly placed somata in the inner nuclear layer (with only occasional displaced cells), (ii) the sparse branching and long wide-field dendritic morphology, and (iii) the co-stratification of AC dendrites with dendrites of injected RGCs in both the NB and the serotonin injections (also co-stratifying with HCN4-positive axonal terminals of type 3a bipolar cells).

### 3.5 The consecutive labeling of neighbor OFF alpha cell arrays

**Figure 5:**
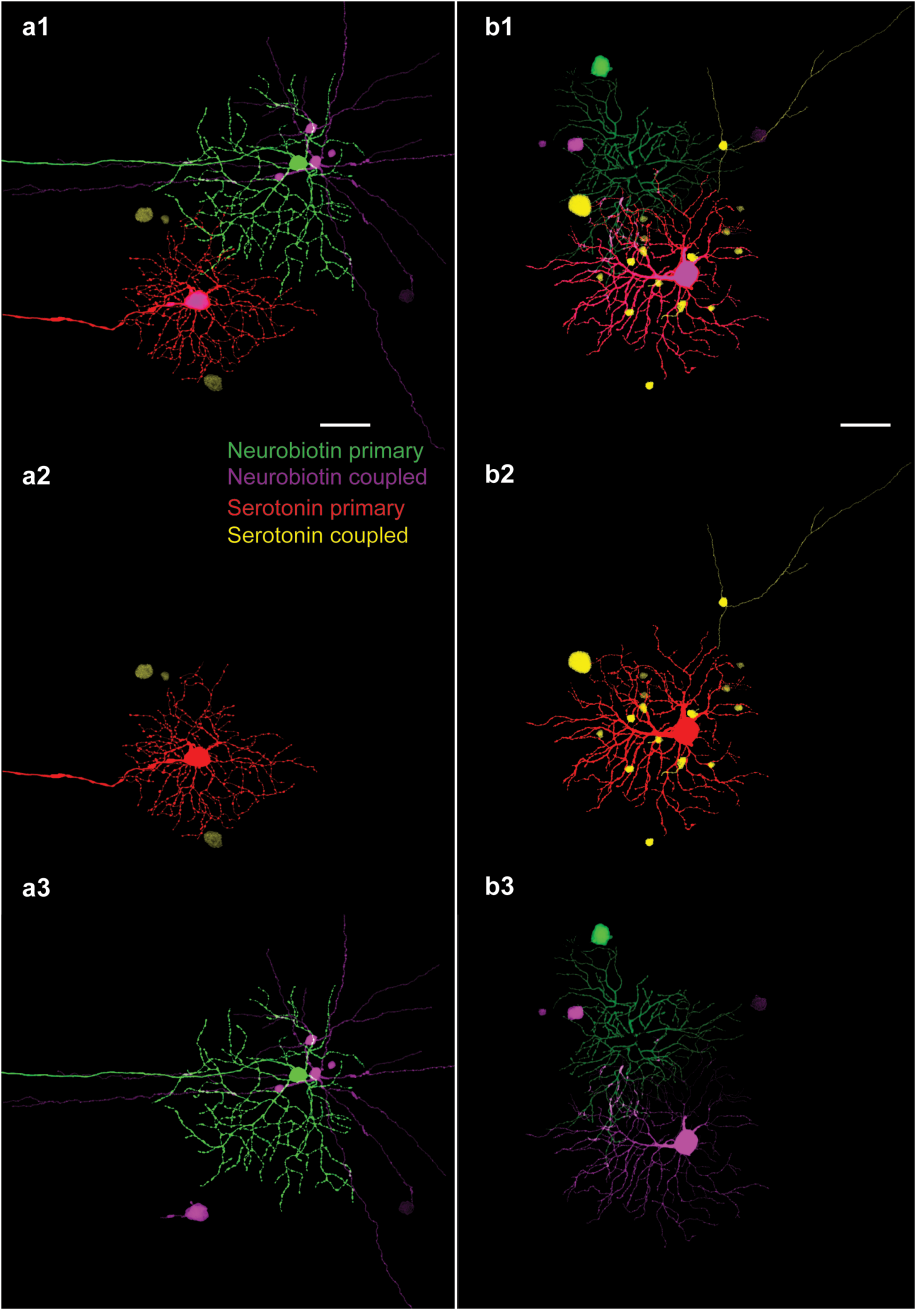
Dual OFF Alpha RGC serotonin/NB injections. **(A)** 1–3. Image set showing the result of parallel injections where one alpha cell was injected with NB (green: NB primary) and one of its direct neighbors was injected with serotonin (red: serotonin primary). As in the case of previous injections, both serotonin- and NB-coupled arrays displayed tracer-coupled ACs (purple: NB coupled; yellow: serotonin coupled). Whereas panel a1 shows the two neighboring OFF alpha RGC arrays together in the same frame, panels a2 and a3 show the isolated serotonin- and NB-coupled arrays, respectively. This consecutive injection of the two tracers did not result in the accumulation of both serotonin and NB tracers in the same ACs. Note that NB, in addition to diffusing into nearby ACs, also diffused into the serotonin-injected RGCs (large purple soma in the middle-bottom of panel a3). **(B)** 1–3. Image set showing the result of another paired injection in which serotonin and NB were injected into the somata of two neighboring OFF alpha RGCs. Whereas NB diffused into the serotonin-injected RGC soma, the cell body of the NB-injected RGC was dislocated as the labeling pipette was pulled back (large green soma in the top left corner), impeding the transcellular diffusion of serotonin into this cell. Although the NB injection was more successful in panel **(A)** serotonin injection was better in panel **(B)**, but neither of the two injections resulted in dually stained coupled ACs. Scale bar: 50 μm.

It has been shown that neighbor OFF alpha RGCs correlate their spiking outputs to maintain a population code to represent features of the visual scene (14, 15, 21, 29–34). It has also been indicated that various forms of these RGC spike correlations are gap junction dependent, as spike synchrony is disrupted by a pharmacological blockade of electrical synaptic transneuronal communication (14, 15, 34). In addition, the ablation of Cx36 gap junctions also deletes both spike synchronization and tracer coupling of RGCs, including those of OFF alpha RGCs. This indicated that RGCs form homologous gap junctions that allow for the two-directional transfer of information and material (e.g., tracer) between neighboring cells. However, it has never been directly determined whether RGC–RGC gap junctions also allow for the intercellular flow of tracer molecules in both directions or whether, on the contrary, some rectification takes place. In the above experiments, we found that serotonin can be utilized to replace NB in tracer injections and that it provides a cellular label comparable to those of classical NB injections. Therefore, these findings allowed us to perform dual tracer injections in direct neighbor OFF alpha RGC pairs (*n* = 3) under the epifluorescent view of the physiological microscope (**Figures 5A, B** show two such dually labeled OFF alpha RGC pairs). In these experiments, one OFF alpha cell was injected with NB, whereas its neighbor was injected with serotonin. We found that it was feasible to carry out such dual alpha RGC pair injections, and both injected RGCs were revealed by the tracers. In addition, the above-described serotonin and NB coupling patterns were revealed. However, we experienced some difficulty with these latter paired injections as either one of the labels (the serotonin or the NB injection) showed subnormal tracer coupling. At this point, it is uncertain whether this failure was due to the small number of repetitions or whether it was the result of tracer interference mechanisms similar to those described above. In fact, we found that regardless of the tracers, it was always the first tracer injection that revealed the well-stained coupled array, while the second tracer injection was less effective. In addition, NB always accumulated in the soma of serotonin-injected RGCs, showing that this putative interference is not complete and gap junctions between the neighboring alpha cells are still conductive.

## 4 Discussion

In this study, we utilized two canonical neuronal dyes, Alexa568 and NB, to mark selected OFF alpha RGCs to reveal their soma/dendritic morphology and to characterize their electrically coupled arrays as well as tracer diffusion. In addition, we included serotonin in our new investigation to test if this newly recognized gap junction-permeable tracer can be utilized in *in vitro* retina tissue, particularly our recent target cell, the OFF alpha RGC.

### 4.1 Serotonin is a potent transjunctional tracer in the *in vitro* tissue

Hou and colleagues (10) have recently shown that, by virtue of its low molecular weight, serotonin can be utilized as a transjunctional tracer in experiments, either supplementing NB or substituting for it. According to the authors, serotonin passed through gap junctions formed between cultured HeLa cells. This serotonin coupling, in conjunction with NB coupling, was eliminated pharmacologically by canonical gap junction blockers. We found that a similar serotonin injection into our target OFF alpha RGC resulted in a successful intercellular coupling and revealed a corresponding gap junction coupled array. This serotonin coupling, in fact, resembled those seen in NB-injected OFF alpha cells, displaying both coupled neighboring alpha cells and a cohort of nearby ACs. Thus, for the first time, we showed that serotonin is a potent neuronal tracer in *in vitro* tissue experiments. The original mouse OFF alpha cell gap junction coupling descriptions (11, 12) reported two sets of NB-coupled AC arrays, one set with larger round somata and a second set with smaller, lightly labeled somata reminiscent of those we show here. The existence of the same two, dim and bright, coupled AC populations was evident following injections of serotonin tracer. The great similarity of serotonin- and NB-injected OFF alpha cell arrays observed was somewhat surprising to us. Although the molecular weight of serotonin is lower than those of NB, their water solubilities were very different. NB is hydrophilic and dissolves easily; however, we had to make extra efforts to dissolve serotonin in the internal solution and to keep it dissolved to perform intracellular injections. This is in line with reports describing the water solubility of these two molecules: NB (typically used in 2%–4% solution) is water soluble up to 10% (100 mg/mL, Vector Laboratories), whereas the water solubility of serotonin is considerably lower (17 mg/mL, Sigma-Aldrich; note that the greater water solubility of NB reflects the fact that a higher concentration of tracer can be used for injections, thus potentially increasing the sensitivity of the *post hoc* histology). Therefore, our observations indicate that solubility in water is not among the factors that significantly determine gap junction permeability. However, a clear conclusion regarding the correlation of water solubility and gap junction permeability can only be drawn after a thorough examination of several molecular compounds, and Mills and Massey (7) performed a meticulous line of experiments to show how molecular weight affects the transjunctional conductance of various biotinylated tracers.

### 4.2 Interference of tracer labels in the OFF alpha RGC array

We concluded that tracers injected in parallel interfere with each other. This conclusion was based on several observations. First, we failed to obtain acceptable tracer coupling in experiments in which we co-injected serotonin and NB from the same pipette. In these experiments, most often, one or the other tracer labeled the injected cell only (or just the soma), although a quantitative analysis was not performed. This somewhat contradicts the findings of Hou and colleagues (10), who found that both serotonin and NB successfully passed through HeLa cell gap junctions. However, there are two major differences between our work and the previous study by Hou and colleagues (10). Whereas Hou and colleagues performed their tests in cell cultures, our investigations were carried out in tissue *in vitro*, where conditions were closer to physiological. In addition, the HeLa cells in the experiments by Hou and colleagues maintained gap junctions constituted partially by Cx43 subunits, which have been shown to provide high transjunctional conductivity [2 μS (35)], whereas the conductance of Cx36 gap junctions in OFF alpha RGC arrays in the rate retina is ≈1,000× lower [1.35 nS (17)]. This discrepancy in gap junction conductance may result in a dissimilar transjunctional tracer diffusion in these two gap junction-coupled systems when tracers are co-injected. A second observation involved NB and Alexa568 injected from the same glass pipette. Alexa dyes are generally used (including in our laboratory work) with NB in the same internal solution, with successful staining of coupled RGC arrays. Therefore, the interference of Alexa568 with NB is not as severe as what we observed when using serotonin and NB together. Nevertheless, when we compared labels of both NB and Alexa568 (0.5%) with those injected with NB only, we observed a clear quantitative difference. When NB was applied alone, both the labeling intensity of individual cells and the number of coupled cells with P were greater than in cells injected with the mixture of NB and Alexa568. The background of this labeling interference is yet unknown, but the two compounds have a somewhat different overall charge, as NB is positively charged (36), whereas the Alexa dyes are negatively charged (37). For this reason, the two compounds require opposite polarity currents to iontophorize targeted neurons, and thus, a certain current polarity needed for one of the compounds may impede the diffusion of the other. In addition, it is also possible that the two oppositely charged molecules electrostatically attract each other or form transient molecular assemblies (i.e., pairs or larger conglomerates), thereby impeding the diffusion of both compounds. A similar tracer interference has been observed when positively charged NB and negatively charged Lucifer Yellow are used in combination in the same injection (previous observations, not shown here). Therefore, such tracer interference must be considered when future tracer labeling experiments are designed.

### 4.3 OFF alpha RGC functionality

In this study, OFF alpha RGCs were used as a model system to describe serotonin as a potential gap junction-permeable tracer in the *in vitro* retina. We performed a morphometric analysis in the somata of both serotonin- and NB-coupled ACs to provide quantitative evidence of the existence of the two AC populations and to find any selectivity of our tracers (NB and serotonin) to preferentially stain either of these populations. The results of this analysis failed to separate the ACs into two (or more) populations, as the available morphological features showed a continuum of values. Our quantitative analysis resulted in two main findings. First, we not only found that serotonin-injected OFF alpha RGCs were reminiscent of their NB counterparts, but also that the coupled arrays shared most quantitative measures, including the same type (based on AC soma parameters) and number of coupled cells and similar staining intensities, and showed processes of the same coupled ACs. Second, using the morphological features in our analysis, fluorescence intensity was the only feature that separated tracer-coupled AC somata into two subpopulations in both the NB-injected and serotonin-injected alpha RGCs. Although coupled ACs with bright somata often displayed labeled dendritic processes that clearly identified them as wide-field ACs, the identity of the dimmer coupled cells that showed no visible processes remains unknown. This second cohort of coupled ACs shares many morphological features with the previous wide-field cells, including soma size and shape (round). This suggests that the two populations are members of the same AC subtype and that the differential labeling intensity is the result of some other factor that determines the amount of tracer flowing through gap junctions from the injected RGC. However, the separation of the two populations is robust in the frequency histograms, and this separation is apparent in both cohorts of tracer injections (i.e., serotonin and NB). Therefore, based on these seemingly contradicting observations, we cannot draw a clear conclusion and leave it to future work to reveal whether the two AC populations are members of two distinct subtypes with many morphological features, or whether they belong to the same cell type and bright and dim cells represent two different functional states. In this latter case, gap junctions of the dim and bright AC populations are in low and high transjunctional conductance states, respectively.

## Data availability statement

The raw data supporting the conclusions of this article will be made available by the authors, without undue reservation.

## Ethics statement

The animal study was reviewed and approved by the ethics committee of the University of Pécs.

## Author contributions

Conceptualization, BV and GS; methodology, GS; validation, BV and GS; formal analysis, GS; investigation, GS, TK-Ö, and GH; resources, BV; data curation, GS, TK-Ö, and GH; writing—original draft preparation, BV and GS; writing—review and editing, BV, GS, TK-Ö, and GH; visualization, GS and BV; funding acquisition, BV and GS. All authors contributed to the article and approved the submitted version.
